# Application of the Socio-Ecological Model to predict physical activity behaviour among Nigerian University students

**DOI:** 10.11604/pamj.2017.26.110.10409

**Published:** 2017-03-01

**Authors:** Inimfon Aniema Essiet, Anisah Baharom, Hayati Kadir Shahar, Benjamin Uzochukwu

**Affiliations:** 1 Department of Community Health, Faculty of Medicine and Health Sciences, University Putra Malaysia (UPM), Selangor, Malaysia; 2 Department of Community Medicine, College of Medicine, University of Nigeria, Enugu Campus, Nigeria

**Keywords:** Social-ecological model, physical activity, Nigeria

## Abstract

**Introduction:**

Physical activity among university students is a catalyst for habitual physical activity in adulthood. Physical activity has many health benefits besides the improvement in academic performance. The present study assessed the predictors of physical activity among Nigerian university students using the Social Ecological Model (SEM).

**Methods:**

This cross-sectional study recruited first-year undergraduate students in the University of Uyo, Nigeria by multistage sampling. The International Physical Activity Questionnaire (IPAQ) short-version was used to assess physical activity in the study. Factors were categorised according to the Socio-Ecological Model which consisted of individual, social environment, physical environment and policy level. Data was analysed using the IBM SPSS statistical software, version 22. Simple and multiple logistic regression were used to determine the predictors of sufficient physical activity.

**Results:**

A total of 342 respondents completed the study questionnaire. Majority of the respondents (93.6%) reported sufficient physical activity at 7-day recall. Multivariate analysis revealed that respondents belonging to the Ibibio ethnic group were about four times more likely to be sufficiently active compared to those who belonged to the other ethnic groups (AOR = 3.725, 95% CI = 1.383 to 10.032). Also, participants who had a normal weight were about four times more likely to be physically active compared to those who were underweight (AOR = 4.268, 95% CI = 1.323 to 13.772).

**Conclusion:**

This study concluded that there was sufficient physical activity levels among respondents. It is suggested that emphasis be given to implementing interventions aimed at sustaining sufficient levels of physical activity among students.

## Introduction

Researches have shown that habitual participation in physical activity (PA) decreases the risk of chronic diseases and conditions including Type II diabetes, cardiovascular diseases, certain types of cancer and obesity [1, 2]. It has also been documented that physical activity helps in building and maintaining healthy bone and muscle and is effective in reducing feelings of depression [2, 3]. The current guidelines by World Health Organization (WHO) recommend that adults aged 18-64 should perform nothing less than 150 minutes of moderate-intensity aerobic physical activity in a week or a minimum of 75 minutes of vigorous-intensity physical activity per week to achieve these health benefits [4].

However, despite these health-enhancing benefits, many studies providing cross-sectional and longitudinal evidence regarding patterns of physical activity have reported that university students are not sufficiently active enough to experience all the potential benefits of physical activity [5–9]. In addition, there is evidence that a larger proportion of students moving to the university engage in low levels of physical activity, with about one-third of those who were previously active, becoming inactive or less active during this transition [10]. However, the decision to engage in an active lifestyle is influenced by various factors such as personal, social and environmental factors [2]. Until very recently, researches on physical activity behaviour has focused mainly on identifying individual determinants [11] and has undermined the impact of the social and physical environment on physical activity behaviour [12]. This approach has been criticised because it places too much emphasis on the individual and does not examine the environment within which the health behaviour occurs [11, 13]. An all-inclusive focus on the factors that determine health behaviour is in tandem with a socio-ecological perspective of human behaviour which suggests the interdependence between people, their behaviour, and their social and physical environment [13].

Social-ecological models provide an overarching framework for understanding the impediments and enablers to physical activity behaviour as it not only focuses on individual characteristics but also considers the social and physical environment context which can include family, friends, neighbourhood associates, formal and informal organizations, design of urban environment and facilities which promote or prevent physical activity [11, 12]. Thus, a social-ecological approach to understanding the influences on physical activity allows researchers to identify vast opportunities to foster the adoption and maintenance of health-enhancing physical activity behaviour, rather than having a traditional isolated focus on intrapersonal factors [12]. Till date, however, there is a lack of published studies which examined the determinants of physical activity participation using the socio-ecological model among Nigerian university students. Hence, the purpose of this study was to investigate the determinants of physical activity practice using the socio-ecological model among a sample of Nigerian university students.

## Methods

**Study design and setting:** The study was a cross-sectional survey, conducted among first-year students of the University of Uyo in Nigeria.

**Sample selection and data collection procedures:** Sample size was calculated using the two-proportion formula by Lemeshow et al. [**14**], taking into consideration non-response and eligibility of respondents. Multistage random sampling was then used to select the respondents for the study. For the first stage of sampling, two faculties (Arts and Social sciences) were randomly selected from the 12 faculties in the university using a computer generated table of random numbers. Thereafter, the students list from the two randomly selected faculties was obtained and 386 first year undergraduate students were selected by stratified (by departments) random sampling proportionate to size using a computer generated table of random numbers.

**Ethical considerations:** Ethical approval was obtained from the University Ethics Committee for Research involving Human Subjects in University Putra Malaysia (JKEUPM). Approval was also obtained from the Deans of the faculties of Arts and Social Sciences in University of Uyo, Akwa Ibom State, Nigeria. Written consent was obtained from each participant before data collection.

**Study variables and instruments:** The dependent variable was the measurement of physical activity. Whilst, the independent variables were according to the socio-ecological model by Stokols [13]. The variables were categorised into individual, social environment, physical environment and policy level factors. The self-administered questionnaire consisted of sections related to these variables.

**Physical activity:** Physical activity behaviour was assessed using the English version of the short self-administered International Physical Activity Questionnaire (IPAQ) (available at http://www.ipaq.ki.se/scoring.pdf) [15]. This short version of IPAQ was developed solely for use among youths and middle-aged adults. It has an acceptable test-retest reliability and criterion validity [16]. The instrument enquires about the number of days, hours and minutes on which the participants performed vigorous physical activity, moderate physical activity (not inclusive of walking) and walking during the previous seven days, from the day of the survey. Activity pattern can be obtained by treating these three activity categories individually. In addition, the total amount of physical activity performed in a week can be estimated by multiplying each activity category by their estimated intensity in METs and then summing the values obtained for the three categories together [15]. A MET represents the amount of oxygen consumed at rest (while sitting quietly at rest). It is equal to 3.5 ml O2/kg/min or≈ 250 mL/min of oxygen consumed and this is the average value for a standard 70kg person [17]. In this study, the MET intensities used to score IPAQ were eight METs for vigorous, four METs for moderate, and 3.3 METs for walking [15]. The outcome variable, physical activity level in the last seven days, was initially categorized into three groups (low, moderate or highly active) as defined by the IPAQ core group [15] as follows: Low-reporting no activity or little activity but not enough to meet the requirements of the other activity categories; Moderate-meeting any of the following three criteria: (a) performing vigorous-intensity activity on three or more days for a minimum of 20 minutes each day, (b) performing moderate intensity activity or waking on five or more days for a minimum of 30 minutes per day, or (c) performing any combination of walking, moderate intensity, or vigorous-intensity activities on five or more days and achieving at least 600 MET-minutes in a week; High-meeting either of the following two criteria: (a) performing vigorous-intensity activity on three or more days and achieving a minimum of 1500 MET-minutes in a week or (b) performing any combination of walking or moderate- or vigorous intensity activities in seven days of a week and accumulating at least 3000 MET-minutes per week. To obtain a dichotomous outcome variable, these three groups were then categorized as insufficient PA or sufficient PA. Sufficient PA was defined as achieving a minimum of 600 MET minutes/week. Hence, they were made up of respondents in the moderate and high intensity categories as they meet the WHO physical activity recommendation for adults aged 18-64 years.

### Individual level factors

**Socio-demography and biological characteristics:** This section consisted of demographic and personal information which include age, sex, ethnicity, marital status and place of residence. Height and weight measurements were also included to calculate the body mass index (BMI). Height and weight measurements were carried out using Seca 213 portable stadiometers and Omron HN-289 digital weight scales respectively.

**Psychosocial variables:** Psychosocial variables consisted of self-efficacy for PA, knowledge, attitude, and perceived barriers. Self-efficacy for PA was measured using the self-efficacy scale developed by Sallis [18]. This tool assesses confidence in one's ability to be physically active under all circumstances. Responses were scored on a 5-point Likert scale ranging from “strongly disagree” to “strongly agree”. An example item was, “I stick to my physical activity program even when I have excessive demands at school”. Continuous scores for self-efficacy were then categorized into two groups (low and high self-efficacy) based on a criteria provided by Leslie et al. [19]. Respondents whose average score was less than or equal to three were regarded as having low self-efficacy while those whose average score was greater than three were regarded as having high self-efficacy regarding physical activity. Knowledge was assessed using the knowledge scale developed by Ward [20] which was based on the guidelines by the American College of Sports Medicine (ACSM). The questionnaire consisted of 10 true-false statements. Responses were scored by allocating one-point to correct answer and zero for wrong answer. An example item was, “Depression and anxiety can be increased by physical activity”. Attitude regarding physical activity was measured using the attitude scale developed by Motl et al. [21]. Responses were scored on a 5-point Likert scale ranging from “strongly disagree” to “strongly agree”. An example item was, “If I am physically active on most days, it would make me better in sports, dance and other activities”. Continuous attitude scores were used for data analysis. Higher scores implied more positive attitude towards physical activity. Perceived barriers was assessed using the barrier subscale of the Exercise Benefits/Barriers Scale (EBBS) questionnaire by Sechrist et al [22]. The tool is used to determine individuals' perceptions regarding the benefits of and barriers to participating in exercise. Responses were scored on a 4-point Likert scale ranging from “strongly disagree [1], disagree [2], agree [3], strongly agree [4]”. An example item was, “Exercise takes too much of my time”. Continuous perceived barrier scores were used for data analysis. Higher scores were indicative of higher perceived barriers towards physical activity.

**Social environment level factors:** Social environment factors consisted of perceived family social support, perceived friends' social support and relatives' physical activity practice. Perceived family and friends' social support for PA was measured using the family and friends social support scales developed by Sallis et al. [23]. These tools measure the influence of family and friends on exercise behaviour. Responses were scored on a 4-point Likert scale ranging from “never” to “regularly”. An example item was, “My family gives me rewards for being physically active”. Continuous scores for perceived family and friends' social support were then categorized into two groups (low and high) based on a criteria provided by Leslie et al. [19] such that respondents whose average score was less than or equal to two were regarded as having low family or friends' social support while those whose average score was greater than two were regarded as having high family or friends' social support regarding physical activity. Relatives (father's, mother's and sibling(s)) PA practice was measured using a single item measure developed by Graham et al. [24]. Participants were asked if their father, mother and sibling(s) are physically active in their free time.

**Physical environment level factors:** There were four items in this section: availability of school facilities for indoor recreation, availability of school facilities for outdoor recreation, perceived safety and enjoyable scenery. Availability of school facilities for indoor and outdoor recreation was measured using a single item measure developed by Graham et al. [24]. Participants were asked if there were school facilities for indoor and outdoor recreation. Perceived safety was measured using a single item measure developed by Paxton et al. [25]. Participants were asked if they perceived that the school was safe for physical activity such as walking, jogging or riding a bike. Enjoyable scenery was measured using a single item measure developed by Brownson et al. [26]. Participants were asked if they perceived that the school had an enjoyable scenery.

**Policy level factors:** Perception on physical education classes and provision of time for physical activity was measured using a single item measure developed by Brownson et al. [26]. Participants were asked if they were mandated to take physical education classes and if the school provided time for physical activity.

**Statistical analyses :** Data was analysed using IBM SPSS version 22.0 (SPSS Inc. Chicago, IL, USA). Descriptive statistics of frequency and percentages were used to determine the prevalence rates for the physical activity categories. Physical activity level as dependent variable was dichotomized as insufficient and sufficient PA. The associations between individual level factors, social environment level factors, physical environment level factors, policy level factors and physical activity were determined using logistic regression with crude odds ratio (OR) at 95% Confidence Interval (CI). Multiple logistic regression with adjusted odd ratio (AOR) and 95% Confidence Interval (CI) were calculated to determine the independent predictors of physical activity. Alpha level was set at 0.05.

## Results

A total of three hundred and forty two respondents completed the survey. The response rate was 88.6%. Of these, 51.8% were males while 48.2% were females. The mean age was 20.67 (S.D ± 3.213). Respondents who were less than 21 years of age constituted 57.3% of the total respondents. The Ibibio ethnic group predominated (70.2%) and almost all the respondents (98%) were single (Table 1). Table 2 shows the distribution of respondents by physical activity level. Mean MET-minutes was 4449.92 (SD ± 3187.45) and median MET-minutes was 3999.75. Based on the operational definition, 93.6% of the participants had done a sufficient amount of physical activity (respondents whose physical activity level was above 600 Met-minutes/week) during the previous seven days from the day of the survey. On univariate analysis, belonging to Ibibio ethnic group, having a normal body mass index, having high self-efficacy, perceived barriers, having sibling(s) that are physically active and availability of school facilities for outdoor recreation were significantly associated with sufficient PA (Table 3, Table 4). On multivariate analysis, participants who belonged to Ibibio ethnic group were about four times more likely to be sufficiently active compared to those who belonged to the 'other' ethnic groups (AOR = 3.725, 95% CI = 1.383 to 10.032). Participants who had a normal body mass index were about four times more likely to be physically active compared to those that were underweight (AOR = 4.268, 95% CI = 1.33 to 13.772) (Table 5).

**Table 1:** Distribution of respondents by socio-demographic characteristics (n=342)

Variable	Frequency	Percentage (%)
**Gender**		
Male	177	51.8
Female	165	48.2
**Age (Years)**		
**Mean (SD)**	20.67 (±3.213)	
<21	196	57.3
≥21	146	42.7
**Ethnicity**		
Ibibio	240	70.2
Others	102	29.8
**Marital status**		
Single	335	98.0
Married	7	2.0
**Place of residence**		
Rural	42	12.3
Urban	300	87.7

**Table 2:** Distribution of respondents by physical activity levels (n =342)

Physical activity (min/week)	Mean (±SD)	Mean number of days/week	Median	IQR	N	%
total met	4449.92 (3187.45)		3999.75	4407		
met						
Vigorous PA	1397.59 (1923.43)	2.47	820	1710		
Moderate PA	1337.09 (1480.33)	4.09	840	1440		
Walking PA	1715.24 (1515.68)	6.05	1039	2508		
level of physical activity						
Low					22	6.4
Moderate					122	35.7
High					198	57.9
physical activity status						
Insufficiently physically active					22	6.4
Sufficiently physically active					320	93.6

**Table 3:** Simple logistic regression showing association between individual, social, physical environment and policy variables and sufficient physical activity (n=342)

Variable	n	f (%)	Crude OR	95% Confidence interval (CI)	p-value
**Age group (years)**					0.059
<21	196	57.3	1.000		
≥21	146	42.7	2.678	0.965-7.437	
**Gender**					0.542
Male	177	51.8	1.000		
Female	165	48.2	0.763	0.321-1.818	
**Ethnicity**					
Ibibio	240	70.2	3.066	1.280-7.348	
Others	102	29.8	1.000		
**Marital status**					0.999
Single	335	98.0	1.00		
Married	7	2.0	1615474912	0.000	
Residence					0.639
Urban	42	12.3	1.000		
Rural	300	87.7	1.429	0.322-6.344	
**Body mass index**					0.003^+^
Underweight	38	11.1			
Normal	265	77.5	4.781	1.629-14.034	0.004^+^
Overweight	32	9.4	0.812	0.234-2.820	0.744
Obese	7	2.0	302901537.1	0.000	0.999
**Self-efficacy**					0.047^+^
Low	225	65.8	1.000		
High	117	34.2	3.505	1.015-12.099	
Perceived barriers			0.872	0.763-0.997	0.045^+^
Knowledge			1.153	0.888-1.495	0.285
Attitude			1.084	0.985-1.194	0.099
**Perceived family social support**					0.550
Low	181	52.9	1.000		
High	161	47.1	1.307	0.543-3.144	
**Perceived friends’ social support**					0.161
Low	95	27.8	1.000		
High	247	72.2	1.884	0.777, 4.565	
**Fathers PA practice**					0.456
Yes	227	66.4	1.398	0.579-3.374	
No	115	33.6	1.000		
**Mothers PA practice**					0.372
Yes	217	63.5	1.486	0.623-3.545	
No	125	36.5	1.000		
**Sibling(s) PA practice**					0.047^+^
Yes	275	80.4	2.528		
No	67	19.6	1.000	1.014-6.302	

Keys: OR, Odd Ratio; CI, Confidence Interval

+ p-value < 0.05

| p-value < 0.25

**Table 4:** Simple logistic regression showing association between individual, social, physical environment and policy variables and sufficient physical activity (n=342)

Variable	n	f (%)	Crude OR	95% Confidence interval (CI)	p-value
**Availability of facilities for indoor recreation**					0.008^+^
Yes	231	67.5	3.272		
No	111	32.5	1.000	1.354-7.909	
**Availability of facilities for outdoor recreation**					0.912
Yes	328	95.9	1.125		
No	14	4.1	1.000	0.140-9.014	
**Perceived safety**					0.707
Yes	287	83.9	0.814	0.232-2.850	
No	55	16.1	1.000		
No	55	16.1	1.000		
**Enjoyable scenery**					0.051
Yes	262	76.6	2.428	0.997-5.912	
No	80	23.4	1.000		
**Perception on physical education classes**					0.324
Yes	76	22.2	1.872		
No	266	77.8	1.000	0.539-6.503	
**Perception on provision of time for physical activity**					0.390
Yes	141	41.2	0.684		
No	201	58.8	1.000	0.288-1.625	

**Keys:** OR, Odd Ratio; CI, Confidence Interval

+ p-value < 0.05

| p-value < 0.25

**Table 5:** Multiple logistic regression for predictors of sufficient physical activity (n=342)

Factors	β	S.E	Wald	df	p-value	Adjusted OR	95% CI	
							Lower	Upper
**Age category**								
<21						1.000		
≥21	0.918	0.627	2.147	1	0.143	2.504	0.733	8.552
**Ethnicity**								
Ibibio	1.315	0.505	6.770	1	0.009^+^	3.725	1.383	10.032
Others						1.000		
**Body mass index**								
Underweight			11.093	3	0.011^+^	1.00		
Normal	1.451	0.598	5.895	1	0.015^+^	4.268	1.323	13.772
Overweight	-0.486	0.757	0.412	1	0.521	0.615	0.139	2.712
Obese	18.718	14577.709	0.000	1	0.999	134682302.3	0.000	
**Self-efficacy**								
Low						1.000		
High	0.385	0.703	0.300	1	0.584	1.469	0.371	5.821
Perceived barriers	-0.041	0.080	0.270	1	0.603	0.960	0.821	1.121
Attitude	0.073	0.062	1.409	1	0.235	1.076	0.953	1.215
**Friends’ social support**							3.503	
Low						1.000		
High	0.220	0.527	0.174	1	0.677	1.246	0.443	
**Sibling(s)’ physical activity**							5.340	
Yes	0.541	0.579	0.875	1	0.349	1.718	0.553	
No						1.00		
**Availability of school facilities for indoor recreation**							5.948	
Yes	0.806	0.498	2.615	1	0.106	2.239	0.843	
No						1.00		
**Enjoyable scenery**							7.009	
Yes	0.888	0.540	2.699	1	0.100	2.430	0.842	
No								
Constant	-2.442	2.370	1.062	1	0.303	0.087		

## Discussion

Being physically active especially during the university period is essential to ensure good mental and physical health later on in life [**2**]. This study noted that participation in sufficient levels of physical activity among first year undergraduate university students was very high (93.6%). The prevalence noted in this study is slightly higher than that reported by Awotidebe et al. [27] which was 89.4%. A study by Sreeramareddy et al. [28] however noted a much lower prevalence of sufficient levels of physical activity, i.e. only 51.1%. The difference in physical activity observed in this study when compared to that of Awotidebe et al. [27] and Sreeramareddy et al. [28] may be attributed to the nature of the questionnaire which was self-report. This method of assessment of PA presents a risk of overestimating the physical activity levels of the respondents [27].

Application of the socio-ecological model to determine the factors associated with physical activity could assist in the development of an intervention design which seeks to include a more holistic program to improve physical activity participation among young adults. In the individual level, interestingly, this study found that the physical activity pattern between males and females was similar. This is contrary to the findings of numerous researches which have reported that males tend to be more physically active than females [1, 28, 29]. This could be attributed to the changes in social norms regarding physical activity and traditional gender roles where it is now becoming more acceptable for women to participate in physical activity [1]. It could also be due to the presence of rigorous academic activities within the university which are demanding for both males and females. Ethnicity was associated with sufficient PA with those belonging to the Ibibio ethnic group being more likely to be physically active compared to others (which comprised of Hausa, Yoruba, Efik and Igbo). Similar to these findings, a study by Adegoke et al. [29] observed that undergraduate students of the University of Ibadan in Nigeria who belonged to Hausa ethnic group were less likely to be moderately physically active compared to those that belonged to other ethnic groups (Ibo, Yoruba and Others). Researches aimed at understanding the differences in physical activity among different ethnic and racial groups have identified socioeconomic status and poor living conditions as being partly responsible for the lack of physical activity among different ethnic groups [1]. However, the present study did not investigate the socio-economic status of the respondents. Thus, it is difficult to conclude that the socio-economic status of the respondents played a part in the observed ethnic differences in the present study. Therefore, future research in a broader population is required to fully investigate the influence of ethnicity on physical activity behaviours among Nigerian university students.

Concerning BMI, the present study found that BMI was significantly associated with sufficient PA. Respondents who had a normal BMI were more likely to be physically active compared to their underweight counterparts. This is in line with the findings of previous researches which reported a significant association between physical activity and body mass index [2, 29–31]. One could postulate that physically activity helps in the maintenance of a healthy weight among the students. Furthermore, underweight students may be less involved in sports as a result of their poor energy levels and/or a greater disposition to fatigue [30]. Self-efficacy was also significantly associated with the physical activity levels of respondents in this study. It was observed that respondents who had high self-efficacy were more likely to be physically active compared to those with low self-efficacy. Previous descriptive studies have also reported a significant direct association between physical activity and self-efficacy [32, 33]. Awotidebe et al. [27] observed that high self-efficacy was associated with sufficient PA such that respondents with high self-efficacy were twice more likely to be sufficiently physically active. Yan et al. [34] also reported that there was a significant association between self-efficacy and meeting physical activity recommendations among Chinese international college students. Self-efficacy is a personal attribute which increases self-esteem and confidence in one's ability to task up challenges that may improve healthy lifestyle. However, it was observed that a greater percentage of respondents in this study had a low self-efficacy. It is recommended that the students be given access to information on healthy behaviour which may in turn enhance their confidence in being regularly active in the face of salient barriers.

In the social environment factor level, only perception of sibling(s) PA was significantly related to sufficient PA. Those who perceived that their sibling(s) were physically active, were more likely to be physically active themselves. Similarly, studies by Martín-Matillas et al. [35] found that having an older brother who was currently active significantly increased the probability of being an active adolescent male. He further stated that the probability of being an active adolescent female increased significantly with having an older sister or an older brother who was currently active. In the physical environment factor level, availability of school facilities for indoor recreation (such as indoor swimming pool, gymnasium, dance studio, weight room, and cardio centre) was the only factor associated with sufficient PA. Respondents who reported that there were indoor school facilities for PA were more likely to be physically active. Consistent with this finding is that of Sreeramareddy et al. [28] which found that students who perceived that there were ´some´ or ´many´ physical facilities for PA were likely to have done physical activity at sufficient levels. In addition to having facilities for PA, the general condition of the available facilities is also important for a decision to indulge in PA. Hence, future studies should also take into account the attractiveness, convenience and barriers to the use of exercise facilities.

Multiple logistic regression showed that the independent predictors of PA among respondents in this study were ethnicity and body mass index. This finding is in agreement with that of Adegoke et al. [29] and Khalaf et al. [30]. Ethnicity and body mass index are both factors on the individual level of the socio-ecological model. This finding suggests that the more proximal factors of the model have a greater influence on PA participation among respondents in this study compared to the distal factors. There are a few limitations to this study. Firstly, the study was carried out among students in a University which produced a sample with high educational status, thus reducing the ability to generalize findings to other Nigerian young adults. Future studies should involve samples from a more heterogeneous Nigerian population. Secondly, the method used to assess physical activity level was self-reported/subjective method which is prone to certain biases. This method presents the risk of over-reporting the amount of physical activity performed by the respondents. Thirdly, this study was cross sectional in nature, as such, the determinants of PA from this cross-sectional survey lack temporal sequence thus limiting the assessment of causality in the observed associations in the variables studied. Despite these limitations, the survey may serve as a point of reference for further studies to assess interactions between individual, social environment, physical environment and policy factors. However, future research should involve larger sample groups and utilize objective methods of assessment of physical activity (such as the use of pedometers or accelerometers). Furthermore, further research is also required to identify more factors that influence physical activity participation among Nigerian university students.

## Conclusion

This study shows that majority of surveyed Nigerian undergraduate students engage in physical activity at sufficient levels. The study also identified factors associated with physical activity using the socio-ecological model. This would be useful in the development of interventions which take into account the multi-level influences on physical activity participation among university students. It is therefore recommended that emphasis be given to implementing interventions aimed at sustaining sufficient levels of physical activity among students.

### What is known about this topic

- Physical activity participation declines as students' transition from high school into the university;
- To the knowledge of the authors, no published study has examined physical activity participation among undergraduate students of University of Uyo, Nigeria, using the socio-ecological model.

### What this study adds

- This study provides descriptive data on the prevalence of physical activity among undergraduate students of the University of Uyo, Nigeria;
- The study also identifies factors that have a significant influence on physical activity participation among University students.
